# Case of Strangulation of the Intestine by a Diverticulum Intestini

**Published:** 1853-06

**Authors:** 


*Case of Strangulation of the Intestine by a Diverticulum Intestini: by Dr. Levick, of Philadelphia*—E. W., dentist, æt. 30, had been subject from infancy to attacks of colic, which were generally releived by vomiting. For a long time before his illness he had been costive, his bowels being moved only by injections of cold water. During the last week of his life, he had been scarcely free at any time from griping pains.

He partook of a cold dinner on the 29th, four hours after which he was seized with vomiting, which continuing, Dr. Kite was sent for. He found him suffering great pain and ejecting from his stomach everything taken into it.

Sinapisms and fomentations were applied to the epigastrium, and the patient was placed in a warm bath, but without any appreciable benefit. Opiate injections were the only means employed that afforded any relief. To overcome the obstruction which was believed to exist, five quarts of tepid water were slowly and carefully injected into the bowel.

The vomiting continued with but slight intermission, and the patient died on the following morning at 8 o'clock.

The preceding history of the case was furnished by Dr. Kite, who with Dr. Remington had charge of the patient.

The examination was made by Dr. Levick December 29, in the presence of Drs. Kite, Remington, and Davis, fifteen hours after death.

*Exterior.*—Rigidity complete; much lividity of the body; face discolored by an offensive dark-colored fluid which had escaped from the mouth; abdomen tympanitic; odor of decomposition very great.

The *abdomen* alone was closely examined. The small intestines were of a dark-pnrple hue, and full of gas.

The *cæcum* full of gas, lifted entirely out of the iliac fossæ, and having the appendix vermiformis lying loosely upon it. A little below and to the right side of the umbilicus, there was found a digital band firmly adherent by one extremity to the parietes of the abdomen. Upon examination, this was found to be a cæcal sac or diverticulum coming off from the ileum at a distance (when the intestine was stretched out) of about two feet from the ileocæcal valve, and of a size, at its connection with the intestine, sufficient to admit the little finger; having the same structure and investments as the intestine. This completely encircled the gut, having produced strangulation and gangrene.—*Ibid.*

